# Efficient adsorption performance of uranium in wastewater by novel MXene material TiVCT_*x*_ and its aerogel composites†

**DOI:** 10.1039/d4ra05531d

**Published:** 2024-10-30

**Authors:** Xiaoxia Luo, Xianliang Ren, Hongwei Wang

**Affiliations:** a Chongqing College of Mobile Communication Chongqing 401520 P. R. China xiaoxialuo90@163.com; b National Key Laboratory of Advanced Casting Technologies, Chongqing Key Laboratory of Soft Condensed Matter Physics and Smart Materials, College of Physics, Chongqing University Chongqing 400044 P. R. China 20202701013@cqu.edu.cn; c Department of Ultrasound, Xinqiao Hospital, Army Medical University (Third Military Medical University) Chongqing 400044 P. R. China

## Abstract

This work focuses on the application potential of novel MXene materials in the field of uranium-containing wastewater adsorption, particularly addressing gaps in existing research. Ultra-thin layered TiVCT_*x*_ was selected as the core adsorbent to thoroughly investigate its adsorption performance of uranium(U(vi))-containing wastewater. By compounding with sodium alginate, we successfully prepared easily recoverable aerogel beads and evaluated their adsorption capacity for ultra-low concentrations of U(vi) in seawater. The findings of this study reveal that TiVCT_*x*_ exhibits optimal adsorption capacity for U(vi) in a weakly acidic environment with a pH of 5.59, and its maximum adsorption capacity for U(vi) reaches up to 336 mg g^−1^, demonstrating superior performance when it comes to other MXene materials. Further research reveals that the adsorption mechanism involves the synergistic effect of electrostatic adsorption and reduction adsorption, exhibiting monolayer adsorption characteristics, and the adsorption process is a spontaneous endothermic reaction. Notably, in simulated complex seawater environments, even when the U(vi) concentration is as low as, for instance, 3.3 μg L^−1^, 50 mg of aerogel beads can still achieve an adsorption capacity of 3.89 mg g^−1^ for 60 L of seawater. These findings underscore the outstanding performance of TiVCT_*x*_ as a novel MXene material in U(vi) adsorption and its broad potential for practical applications.

## Abstract 2

This work focuses on the application potential of novel MXene materials in the field of uranium-containing wastewater adsorption, particularly addressing gaps in existing research.

## Graphical abstract

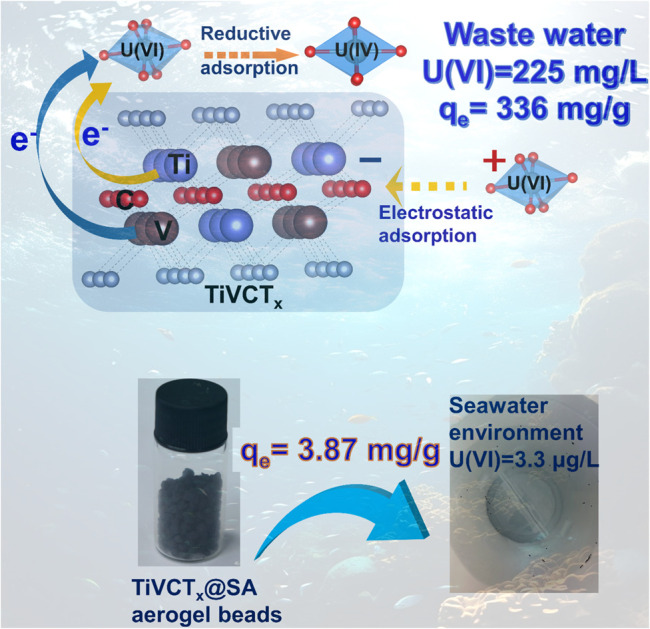

## 1. Introduction

With the ongoing advancements in nuclear energy technology, the issue of nuclear wastewater has become increasingly prominent, and its potential hazards cannot be overlooked.^1,2^ The radioactive substances contained within nuclear wastewater not only have the potential to induce cellular mutations and genetic information damage but also significantly elevate the risk of malignant diseases, including cancer.^3,4^ Even more severe is the fact that, upon infiltrating natural water bodies, these radioactive wastewaters can cause long-term and severe harm to aquatic organisms through the cumulative effects of the food chain, ultimately posing a fatal threat to the entire ecosystem. In addressing the challenges posed by nuclear wastewater treatment, various methods have been widely employed, including precipitation, oxidation–reduction, electrodialysis, bioreduction, and adsorption.^5,6^ Among these, adsorption occupies a significant position in the field of nuclear wastewater treatment due to its multiple advantages, such as high efficiency, flexibility, environmental friendliness, and cost-effectiveness, and it has garnered extensive industrial application.^7^ The tunable pore structure demonstrates extensive application potential in the field of adsorption and separation. As an emerging two-dimensional material, MXene's layered structure, composed of transition metal elements and elements such as carbon and nitrogen, confers upon it remarkable chemical stability and a substantial specific surface area.^8^ The surface of MXene is rich in functional groups such as hydroxyl and oxo groups, providing abundant adsorption active sites. These unique properties endow MXene with significant advantages in the treatment of U(vi)-containing nuclear wastewater.^9^ The adsorption capacity of monolayer Ti_3_C_2_T_*x*_ for U(vi) can reach 214 mg g^−1^,^10^ while that of monolayer Ti_2_CT_*x*_ is even higher, achieving 470 mg g^−1^,^11^ which is 2.2 times that of Ti_3_C_2_T_*x*_. Further modification, such as carboxyl-functionalized Ti_3_C_2_T_*x*_ (TCCH), can enhance its adsorption capacity for U(vi) to 334 mg g^−1^.^12^ Zhao *et al.* made an in-depth study on benzoyl thiourea-fixed activated carbon (BT-AC), and discussed its preparation process and adsorption mechanism for U(vi). The mechanism of BT-AC removal of U(vi) mainly depends on the coordination between U(vi) and the O, S and N atoms in BT-AC. In the environment of competitive ion coexistence, BT-AC can still maintain the selective adsorption of U(vi), and the adsorption capacity reaches 82 mg g^−1^.^13^ Bai *et al.* developed a porous resin (HCR-CO) through structural design of hydroxyphenylacetonitrile modified resin.^14^ The high specific surface area and abundant micropores of HCR-AO can accelerate the diffusion of U(vi) at the outer surface adsorption stage. The maximum adsorption capacity of HCR-AO for U(vi) is 380 mg g^−1^, and the adsorption capacity of HCR-AO is still excellent after 8 cycles. Existing research has fully demonstrated the excellent performance of MXene in U(vi)-containing wastewater treatment. However, current research primarily focuses on the two mature MXene materials, Ti_3_C_2_T_*x*_ and Ti_2_CT_*x*_, with relatively scarce reports on novel MXene, indicating significant research gaps that hinder researchers' deep exploration of MXene's adsorption mechanisms. In fact, over 30 types of MXene have been successfully synthesized,^15^ among which TiVCT_*x*_, as a relatively new M_2_X-type MXene material, shares similar composition and structure with Ti_2_CT_*x*_ and exhibits stronger reduction performance, promising to be a more potential novel adsorbent for nuclear wastewater.^16^ Furthermore, after treating nuclear wastewater as an adsorbent, MXene faces separation challenges, requiring complex separation methods such as vacuum filtration and high-speed centrifugation to achieve effective separation of adsorbed products, which greatly limits its practical application.^17^ Therefore, designing and synthesizing MXene adsorbent materials that are easy to recover and separate is a pressing scientific challenge. In this study, a new MXene-TiVCT_*x*_ with M_2_XT_*x*_ configuration was selected as the U(vi) adsorbent. The doping of V not only makes the MXene of this composition more active, but also provides a reduction adsorption site for U(vi) adsorption, which improves the U(vi) adsorption capacity of MXene. It also provides a new way to develop high performance MXene adsorbents.

In the context of this research, the present study selected the novel MXene material TiVCT_*x*_ as an adsorbent and systematically investigated its adsorption performance and mechanism for U(vi)-containing wastewater. The specific research content encompassed examining the effects of environmental factors such as pH value, temperature, and interfering ions on the adsorption performance of TiVCT_*x*_, and subsequently determining the optimal environmental conditions for its application. Furthermore, this study successfully synthesized TiVCT_*x*_ composite sodium alginate aerogel beads that are easy to recover and separate, which demonstrated high adsorption capacity for extremely low concentrations of U(vi) in simulated seawater environments.

## 2. Experimental

### 2.1 Materials

The precursor TiVAlC was purchased from Beike Nanometer, while uranyl was obtained from Aladdin. HCl, HNO_3_, NaOH, LiF, NaCl, CaCl_2_, KCl, Na_2_SO_4_, and MgSO_4_ were purchased from Sinopharm Chemical Reagent Co., Ltd.

### 2.2 Preparation of TiVCT_*x*_

TiVCT_*x*_ was prepared by etching the precursor TiVAlC with a mixed solution of LiF and HCl. The procedure involved mixing 1.6 g of LiF and 20 mL of 9 M HCl to create the etching solution, adding 1 g of TiVAlC powder, and etching at 45 °C for 48 h. Post-etching, the mixture was washed, ultrasonicated, and centrifuged. The supernatant was collected, yielding the TiVCT_*x*_ solution, which was then freeze-dried to obtain the TiVCT_*x*_ powder.

### 2.3 Preparation of TiVCT_*x*_@SA aerogel

**Fig. 1:**
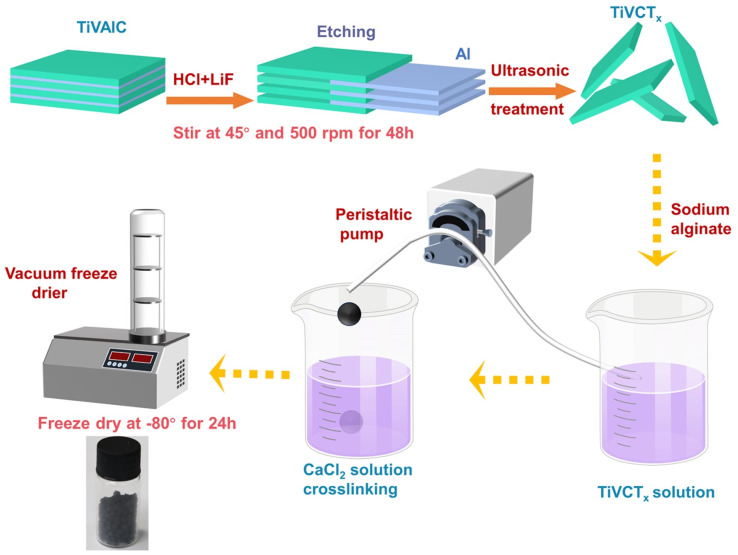
Preparation process of MXene materials and aerogel composites diagram.

A 100 mL solution of TiVCT_*x*_ with a concentration of 4.56 mg mL^−1^ was prepared. Then, 2 g of sodium alginate was added, and the mixture was vigorously stirred for 20 min until a uniform colloidal state was reached. The colloidal mixture was dropwise added into a 2 wt% CaCl_2_ solution and allowed to crosslink for 5 h. After vacuum freeze-drying, the TiVCT_*x*_@SA aerogel was obtained. The preparation process diagram is shown in Fig. 1.

### 2.4 Batch adsorption experiments

U(vi) solutions with varying concentrations were prepared by dissolving uranyl in ultrapure water. During the adsorption process, 20 mg of TiVCT_*x*_ was utilized, with a U(vi) solution volume of 100 mL, under magnetic stirring at a rate of 600 rpm. With the exception of thermodynamic experiments, all experiments were conducted at a controlled temperature of 25 °C. Unless otherwise specified for pH-related experiments, the pH was maintained at the optimal adsorption value of 5. To simulate low-concentration U(vi) adsorption in seawater, the seawater environment was formulated according to the Mocledon synthetic seawater recipe (ESI Table S1†), with a pH of 8.3 and a U(vi) concentration of 3.3 μg L^−1^.

The adsorption capacity and removal efficiency at various time points are determined using eqn (1)–(3):^16^1
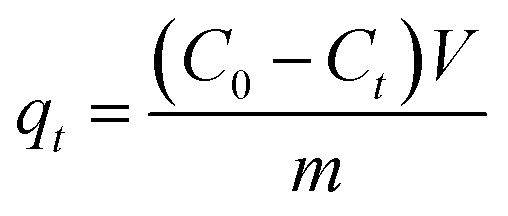
2
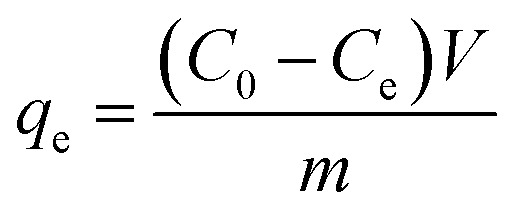
3
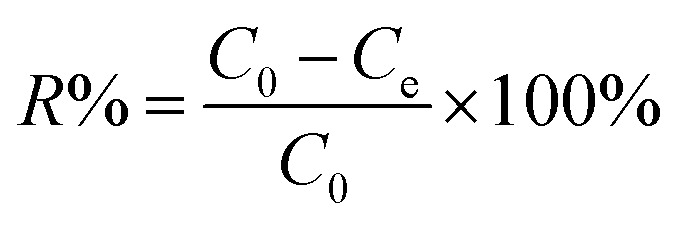


The adsorption capacities (mg g^−1^) at time *t* and equilibrium are denoted as *q*_*t*_ and *q*_e_, respectively. *C*_0_ (mg L^−1^) represents the initial concentration of U(vi), *C*_*t*_ (mg L^−1^) denotes the concentration at time *t*, and *C*_e_ signifies the concentration at equilibrium. *V* denotes the volume (L) of the U(vi) solution, while *m* represents the mass (g) of the adsorbent, and *R* represents the removal efficiency (%).^18^

The fitting formulas of the pseudo-first-order kinetic model, pseudo-second-order kinetic model and Weber diffusion model are as follows:4
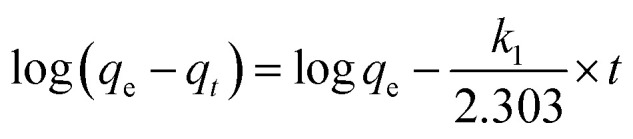
5
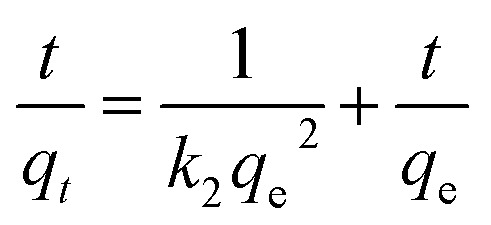
6
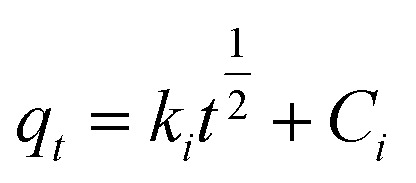
*k*_1_ (min^−1^) and *k*_2_ (g min^−1^ mg^−1^) represent the adsorption rate constants, *k*_*i*_ denotes the coefficient of phase *i* in Weber's model.^19^

The Langmuir and Freundlich isotherm models are derived from the following equations:^20^7
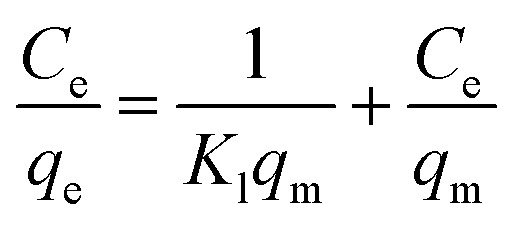
8
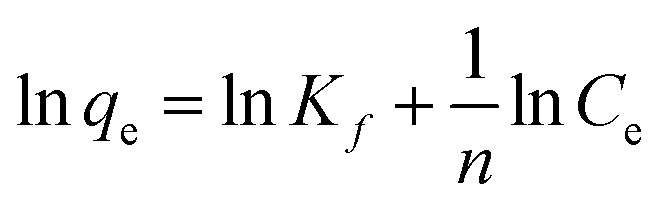
*q*_m_ (mg g^−1^) denotes maximum adsorption capacity; *K*_l_ represents Langmuir constants related to adsorption energy; *K*_f_ is Freundlich model constant; *n* is Freundlich exponent for sorption intensity. Eqn (4)–(8) correspond to a quasi-first-order kinetic model, a quasi-second-order kinetic model, a Weber diffusion model, a Langmuir adsorption isotherm model and a Freundlich adsorption isotherm model.

The formulas for entropy (Δ*S*), enthalpy (Δ*H*), and Gibbs free energy (Δ*G*) are presented below:^21^9
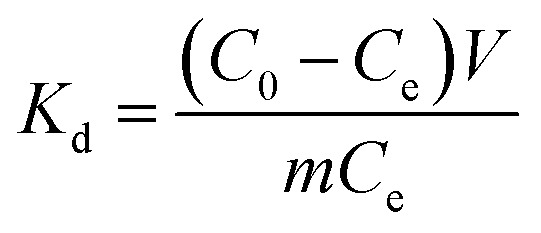
10
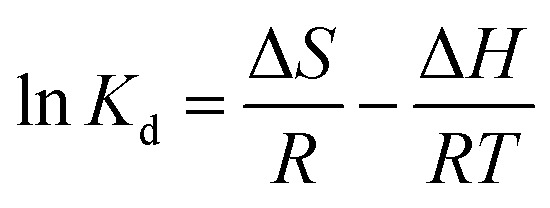

11 Δ*G* = Δ*H* − *T* × Δ*S*

where *K*_d_ is the distribution coefficient (mL g^−1^), *R* stands for the gas rate constant (8.314 J mol^−1^ K^−1^), *T* is temperature in kelvin (K).

### 2.5 Desorption

In this study, a mixture of hydrochloric acid and nitric acid was used as the desorption agent for TiVCT_*x*_. The adsorbed solution was centrifuged at 8000 rpm for 30 min, and the TiVCT_*x*_ nanosheets were separated precipitated and collected. Add 20 mL 5 M HCl and 5 M HNO_3_ into the mixture and stir magnetically at room temperature for 6 h; after washing with deionized water, adjust the pH to 6.5 with 0.1 mol per L NaOH, and then stir for 2 h; the precipitation was obtained by centrifugation at 8000 rpm for 30 min, freeze-dried, and then further adsorbed.

### 2.6 Characterization

X-ray diffractometer (X'Pert Powder X, PANalytical) was used to analyze the crystal structure of the sample, the testing angle is 5–90°, the scanning speed is 2° min^−1^, and the scanning electron microscope (JSM-6800F, JEOL) was used to detect the morphology of the sample. The zeta potential analyzer (Nanobrook Omni, Brookhaven) evaluates the surface charge of the sample, and the X-ray photoelectron spectrometer (ESCALAB 250Xi, Thermo Fisher Scientific) measures the binding energy and valence change of the sample. Ion concentration was measured using a inductively coupled plasma emission spectrometer (PerkinElmer 8300).

## 3. Result and discussion

### 3.1 Characterization of TiVCT_*x*_

**Fig. 2:**
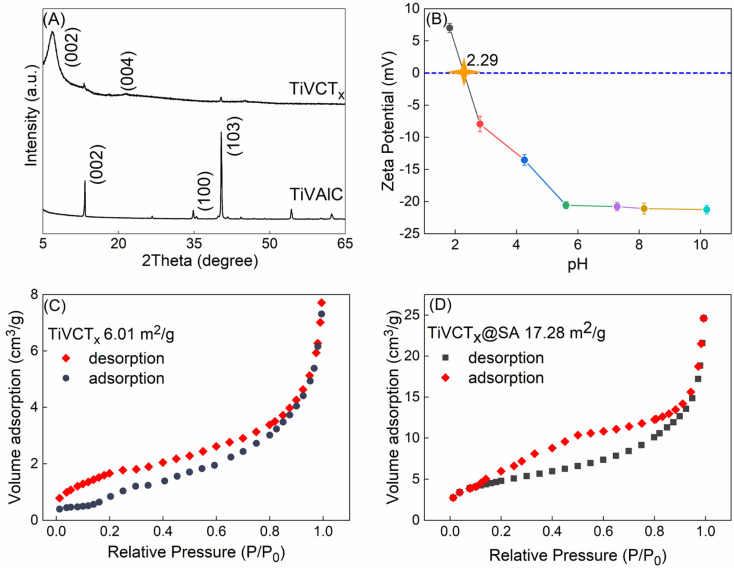
(A) XRD pattern of TiVCT_*x*_; (B) zeta potential diagram of TiVCT_*x*_ at different pH values; (C) N_2_ adsorption–desorption curve of (C) TiVCT_*x*_ and (D) TiVCT_*x*_@SA aerogel.

As depicted in Fig. 2A, the XRD pattern clearly reveals that the diffraction peak corresponding to Al (103) in TiVAlC vanishes completely after the etching process, providing compelling evidence for the successful removal of the Al element. Concurrently, it is observed that the (002) diffraction peak of etched TiVCT_*x*_ shifts towards smaller angles, indicating an increase in lattice spacing as a result of the etching process.^22^Fig. 2B illustrates the variation of the zeta potential of TiVCT_*x*_ under different pH conditions. The point of zero charge (PZC) of TiVCT_*x*_ is 2.29. When the environmental pH is below 2.29, the potential of TiVCT_*x*_ exhibits a positive value; whereas, when the pH exceeds 2.29, its potential shifts to a negative value. Investigating the zeta potential of TiVCT_*x*_ paves the way for a deeper exploration of the mechanism by which pH influences adsorption processes.^23^Fig. 2C and D respectively exhibit the N_2_ adsorption–desorption curves for TiVCT_*x*_ and TiVCT_*x*_@SA aerogel. The BET specific surface area of TiV is found to be 6.01 m^2^ g^−1^, indicating an excellent standing within the MXene family.^24^ By contrast, the BET specific surface area of the TiVCT_*x*_@SA aerogel attains 17.28 m^2^ g^−1^, marking a significant enhancement of more than twofold relative to TiVCT_*x*_. This attribute is particularly conducive to augmenting its adsorption capabilities.

**Fig. 3:**
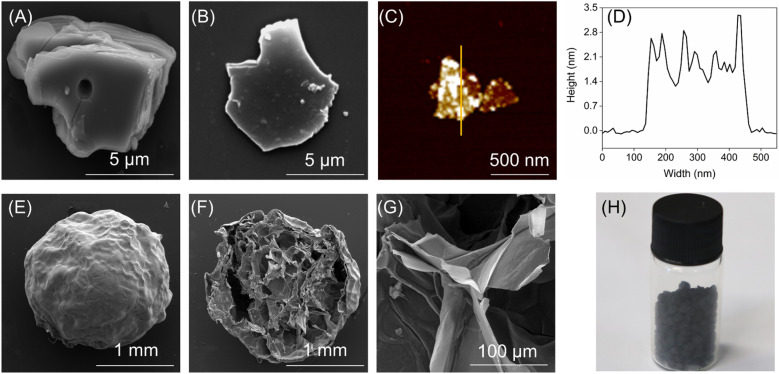
(A) SEM image of TiVAlC; (B) SEM, (C) AFM, and (D) AFM thickness images of TiVCT_*x*_, respectively; (E) SEM, (F) cross-sectional SEM, (G) internal SEM, and (H) physical image of TiVCT_*x*_@SA aerogel.

The SEM images (Fig. 3A and B) showed that the precursor TiVAlC had a massive structure of 14 × 11 μm, while the etched TiVCT_*x*_ had a layered structure of 13 × 10 μm. It is the size of a typical TiVCT_*x*_ slice and is universal, with a small number of smaller TiVCT_*x*_ slices available. Through AFM testing (Fig. 3C), it is determined that the thickness of TiVCT_*x*_ is approximately 2 nm (Fig. 3D), thereby confirming its ultra-thin nanolayer nature. The TiVCT_*x*_@SA aerogel spheres possess a diameter of 1.88 mm, and their cross-sectional SEM image reveals an internal structure reminiscent of a honeycomb, composed of numerous pores (Fig. 3F). Upon further SEM magnification, it is revealed that the interior of these spheres is comprised of thin layered structures (Fig. 3G). Lastly, Fig. 3H provides a visual representation of the TiVCT_*x*_@SA aerogel.

### 3.2 U(vi) adsorption properties of TiVCT_*x*_

#### 3.2.1 Effect of pH

**Fig. 4:**
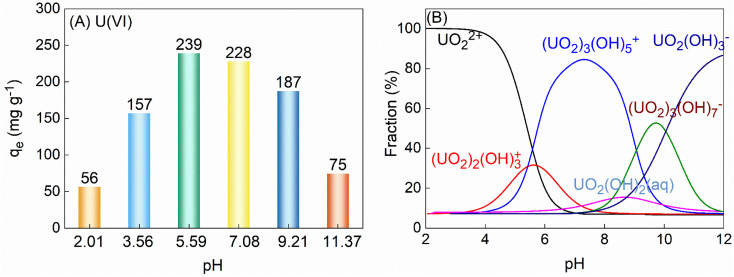
(A) The adsorption capacity of 100 mL 125 mg per L U(vi) adsorbed by 50 mg TiVC varies with pH; (B) the form of U present varies with pH.

To determine the optimal conditions for the application of TiVCT_*x*_ in adsorbing U(vi), this study investigated the adsorption capacity of TiVCT_*x*_ across different pH environments. Initially, pure NaOH was utilized to adjust the pH of the U(vi) solution, effectively eliminating any potential influence of NaOH on the adsorption results (ESI Fig. S1†). As the pH increased from 2 to 11, the adsorption capacity of TiVCT_*x*_ exhibited an initial rise followed by a decline. The maximum adsorption capacity was observed at a pH of 5.59, reaching 239 mg g^−1^ (Fig. 4A). The study further calculated the speciation of U at various pH values, as depicted in Fig. 4B. By analyzing the zeta potential of TiVCT_*x*_ alongside the speciation of U, the mechanism underlying the influence of pH on the adsorption by TiVCT_*x*_ was further elucidated. At pH values below 2.29, TiVCT_*x*_ carries a positive charge and experiences electrostatic repulsion with positively charged UO_2_^2+^, resulting in a low adsorption capacity. Conversely, when the pH exceeds 2.29, TiVCT_*x*_ becomes negatively charged and attracts positively charged UO_2_^2+^*via* electrostatic attraction.^25^ As the negative charge of TiVCT_*x*_ gradually intensifies, the electrostatic attraction strengthens, subsequently leading to an increase in adsorption capacity. However, as the pH continues to rise above 7.1, UO_2_^2+^ combines with a substantial amount of OH and begins to carry a negative charge. At this juncture, the negative charge of TiVCT_*x*_ is nearly maximized, causing electrostatic repulsion between TiVCT_*x*_ and U(vi), which ultimately leads to a decrease in adsorption capacity. Given that the optimal pH environment for the application of TiVCT_*x*_ in adsorbing U(vi) is under weakly acidic conditions, the pH was uniformly set at 5.6 in the subsequent adsorption experiments.

#### 3.2.2 Adsorption kinetics and isotherm

**Fig. 5:**
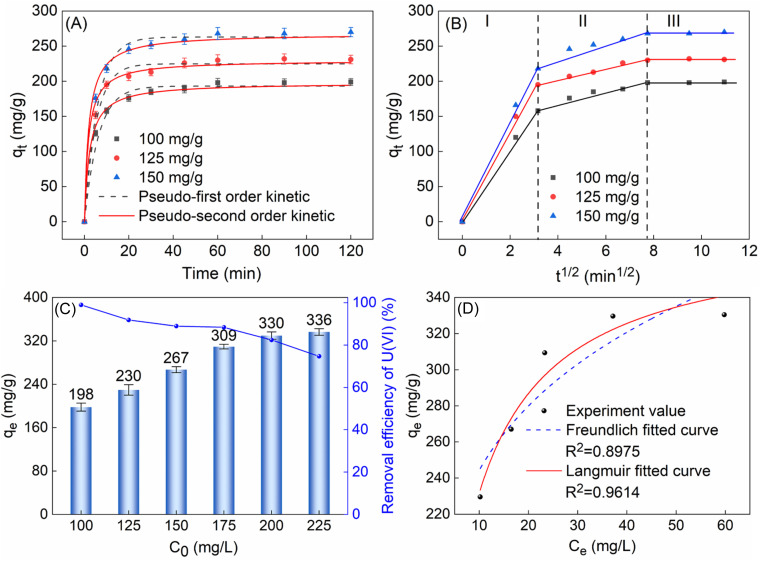
(A) The *q*_*t*_ of 50 mg TiVCT_*x*_ adsorbing 100, 125 and 150 mg per L U(vi) with time; (B) intragranular diffusion model; (C) the *q*_e_ of 50 mg TiVCT_*x*_ varies with the initial concentration of U(vi); (D) Freundlich and Langmuir isotherm fit curves.

To delve into the adsorption process, this study investigated the relationship between adsorption capacity and time. The experimental results indicated that TiVCT_*x*_ achieved over 80% of its equilibrium adsorption capacity within the initial 10 min and reached full adsorption equilibrium at 60 min, with no subsequent increase in adsorption capacity. To further elucidate the adsorption mechanism, pseudo-first-order and pseudo-second-order kinetic models were employed to fit the experimental data (as shown in Fig. 5A). The findings revealed that the pseudo-second-order kinetic model exhibited a correlation coefficient of up to 99% with the experimental data. Moreover, the theoretical equilibrium adsorption capacity derived from this model, calculated using the fitting parameters in Table 1, was in closer agreement with the experimental results. It shows that the adsorption process is more consistent with the quasi-second-order kinetic model, which indicates that the adsorption process is mainly chemisorption.^26^

**Table 1:** Fitting results of PFO kinetics and PSO kinetics

Adsorbate	*C*_0_ (mg L^−1^)	*q*_e e*x*p_ (mg g^−1^)	Pseudo-first-order model			Pseudo-second-order model		
			*q*_e cal_ (mg g^−1^)	*k*_1_	*R* ^2^	*q*_e cal_ (mg g^−1^)	*k*_2_	*R* ^2^
U	100	198	193.32	0.36	0.99	198.69	0.02	0.99
	125	230	225.96	0.43	0.96	229.36	0.02	0.99
	150	267	263.53	0.38	0.95	266.14	0.01	0.98

Furthermore, analysis using the intraparticle diffusion model revealed that the adsorption process consisted of three stages: the first 10 min represented the external diffusion stage, during which U(vi) rapidly diffused to the outer surface of TiVCT_*x*_ and underwent adsorption reactions; the period from 10 to 60 min corresponded to the intraparticle diffusion stage, where, as the outer surface adsorption gradually saturated, U(vi) began to penetrate into the TiVCT_*x*_ layers and undergo intraparticle diffusion and adsorption. After 60 min, adsorption and desorption reached a dynamic equilibrium, resulting in stabilized adsorption capacity. In conclusion, this adsorption process is a heterogeneous diffusion process governed by both external and intraparticle diffusion.^27^

To delve into the adsorption behavior on the surface of TiVCT_*x*_ adsorbent, this study examined its adsorption capacity under different concentrations of U(vi) solutions. The experimental results revealed that as the initial concentration of U(vi) increased, the *q*_e_ gradually augmented. When the concentration reached 225 mg L^−1^, the adsorption capacity tended to saturate and ceased to increase significantly (Fig. 5C). The error bar is the standard error representing the range of error between the sample mean and the population after multiple experimental tests. The maximum adsorption capacity of 50 mg of TiVCT_*x*_ was determined to be 336 mg g^−1^. By fitting the experimental results to both the Freundlich model and the Langmuir model (Fig. 5D), it was found that the Langmuir model exhibited a higher degree of fit, indicating that the adsorption process on the surface of TiVCT_*x*_ was monolayer.^28^

#### 3.2.3 Adsorption thermodynamics

**Fig. 6:**
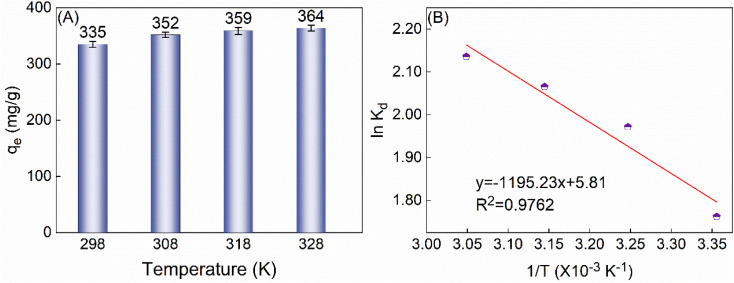
(A) The *q*_e_ of 225 mg per L U(vi) by 50 mg TiVCT_*x*_ varies with temperature; (B) thermodynamic equilibrium constant fitting diagram.

This study investigated the impact of temperature on the adsorption capacity of TiVCT_*x*_ at temperatures of 298, 308, and 318 K. The findings reveal that as the temperature rises, the *q*_e_ gradually increases (Fig. 6A), indicating that the adsorption process is endothermic. Further analysis of the thermodynamic equilibrium constant fitting plot (Fig. 6B) and the relevant thermodynamic parameters derived from the fitting results (Table 2) reveals that the adsorption process exhibits an endothermic nature (Δ*H* > 0) and results in an increase in the system's disorder (Δ*S* > 0).^29^ However, owing to the Gibbs free energy change (Δ*G*) being less than 0, the adsorption process can still proceed spontaneously.

**Table 2:** Thermodynamic parameters of U(vi) adsorption by TiVCT_*x*_

Adsorbate	Δ*H* (kJ mol^−1^)	Δ*S* (J mol^−1^ K^−1^)	Δ*G* (kJ mol^−1^)			
			298 K	308 K	318 K	328 K
U	9.94	48.30	−4.46	−4.94	−5.42	−5.91

### 3.3 Adsorption mechanism

**Fig. 7:**
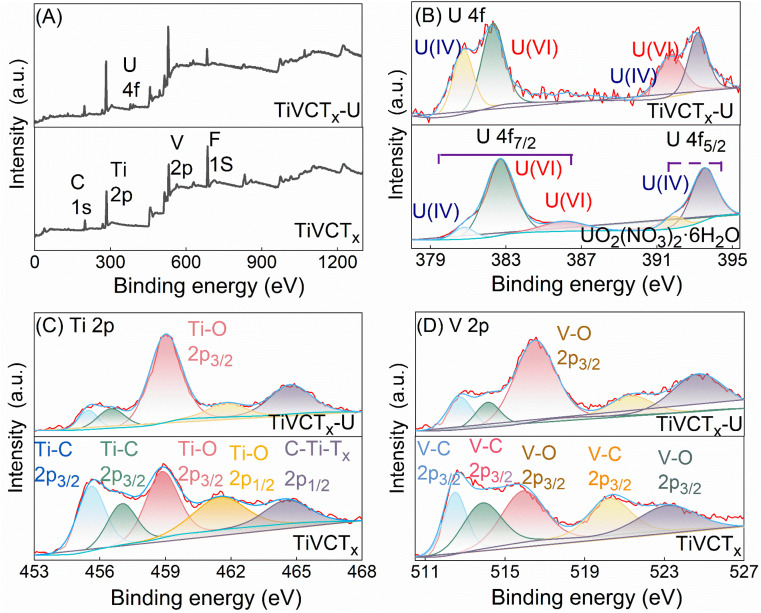
XPS spectra before and after TiVCT_*x*_ adsorbs U(vi): (A) survey scan spectrum, (B) high-resolution U 4f spectrum, (C) high-resolution Ti 2p spectrum, (D) high-resolution V 2p spectrum.

This study analyzed the changes in binding energies of U, Ti, and O elements in TiVCT_*x*_ before and after U(vi) adsorption using XPS tests, and the test results were shown in Fig. 7. The full spectrum scanning results indicated the presence of Ti, C, V, O, and F elements in TiVCT_*x*_ before adsorption, while the emergence of U 4f peaks was observed after adsorption. High-resolution spectra further revealed that the pure uranyl species on the surface of TiVCT_*x*_ before adsorption consisted of U(iv) and U(vi) for both 4f_7/2_ and 4f_5/2_ levels, with U(iv) accounting for 20.75% and U(vi) accounting for 75.25%. After adsorption, the proportions of U(iv) and U(vi) changed to 38.25% and 61.75%, respectively, indicating the reduction of U(vi) during the adsorption process.^28^ Meanwhile, high-resolution spectra of Ti 2p and V 2p showed an increase in the proportions of Ti–O 2p_3/2_ and V–O 2p_3/2_ after U adsorption, suggesting that some Ti and V were oxidized during the adsorption process.^11^ Comprehensive analysis demonstrates that reductive adsorption occurs during the adsorption of U(vi) by TiVCT_*x*_, which has also been reported in other MXenes.^11,30,31^

Based on the combined analysis of zeta potential, adsorption kinetic model fitting, and XPS results, the adsorption mechanism is elucidated as follows: at a pH of approximately 5.59, the surface of TiVCT_*x*_ carries a significant amount of negative charge, facilitating electrostatic adsorption with positively charged U(vi). Simultaneously, a small portion of U(vi) is reduced by unsaturated Ti and V in TiVCT_*x*_, leading to reductive adsorption. This mechanism, which involves both physical and chemical adsorption, confers excellent adsorption capacity to TiVCT_*x*_. As shown in the Table 3, when compared to other MXene adsorbents, TiVCT_*x*_ exhibits a leading adsorption capacity.

**Table 3:** Comparison of adsorption properties between this study and previous reports on MXenes adsorption of U(vi)

Adsorbent	Initial pH	Equilibrium time (min)	Maximum adsorption capacity (mg g^−1^)	References
Ti_3_C_2_T_*x*_	5	150	174	10
Ti_2_CT_*x*_	3	2880	470	11
Ti_3_C_2_T_*x*_-TCCH	4	120	334	12
Ti_3_C_2_T_*x*_@biochar	7	180	239.7	30
LDHs-Ti_3_C_2_T_*x*_	5	180	241	31
Amino-Ti_3_C_2_T_*x*_	5	120	278	32
HPC/Ti_3_C_2_T_*x*_/POSS	5	120	307	33
Carboxyl-Ti_3_C_2_T_*x*_	5.3	120	345	34
TiVCT_*x*_	5.3	60	336	This work

### 3.4 Application of TiVCT_*x*_@SA aerogel in U(vi) adsorption from seawater

**Fig. 8:**
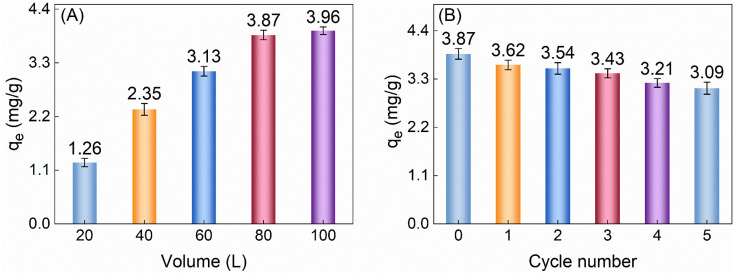
(A) *q*_e_ of 50 mg aerogel for low concentration U(vi) in simulated seawater of different volumes; (B) *q*_e_ of 50 mg for 80 L seawater can be reused.

When investigating the actual adsorption effectiveness of TiVCT_*x*_ towards low-concentration U(vi) in seawater, given the complexity of the marine environment and the low concentration of U(vi) (merely 3.3 μg L^−1^), we prepared easily recoverable TiVCT_*x*_ aerogel beads and applied them to the study of U(vi) adsorption in simulated seawater environments.^35,36^ The experimental results are shown in Fig. 8A. Specifically, in the experiment, 50 mg of aerogel beads were used to adsorb U(vi) from seawater samples of different volumes, each containing 3.3 μg L^−1^ of U(vi). The experimental results indicated that the maximum adsorption capacity of this composite aerogel reached 3.96 mg g^−1^. Furthermore, as shown in Fig. 8B, the aerogel beads demonstrated good recyclability, with their adsorption capacity remaining above 80% of the initial value after five cycles of reuse.

## 4. Conclusion

In this study, an ultrathin nano-layered adsorbent, TiVCT_*x*_, was successfully synthesized, and a simple freeze-drying method was employed to combine it with sodium alginate, resulting in easily recoverable aerogel spheres. TiVCT_*x*_ exhibited a high adsorption capacity for U(vi) in high-concentration wastewater, with a saturation adsorption capacity of 336 mg g^−1^. The adsorption mechanism was thoroughly analyzed, revealing electrostatic adsorption of U(vi) by TiVCT_*x*_ based on zeta potential test results. Additionally, XPS analysis indicated a reduction adsorption mechanism involving the combined action of Ti and V on U(vi). The coexistence of electrostatic and reduction adsorption mechanisms contributed to TiVCT_*x*_'s leading adsorption capacity among MXene materials. Furthermore, in simulated seawater with a U(vi) concentration of only 3.3 μg L^−1^, the TiVCT_*x*_ aerogel spheres demonstrated the ability to adsorb U(vi) from complex environments, achieving an adsorption capacity of 3.96 mg g^−1^. This research not only provides an efficient adsorbent material but also offers important practical insights into the application of MXene in U(vi) adsorption. TiVCT_*x*_ has demonstrated superior adsorption performance and ion interference resistance in experimental settings, indicating its broad potential for the adsorption of U(vi) in seawater. There is significant room for improvement in the application exploration of U(vi) under simulated seawater conditions, and we will continue to delve deeper into this issue in subsequent research.

## Data availability

All data included in this study are available upon request by contact with the corresponding author.

## Conflicts of interest

There are no conflicts to declare.

## Supplementary Material

RA-014-D4RA05531D-s001
