# Supplementary material for: Efficient adsorption performance of uranium in wastewater by novel MXene material TiVCTx and its aerogel composites
Source: RSC Adv. 2024 Oct 30;14(47):34746–55. doi: 10.1039/d4ra05531d (PMC11523290; doi:10.1039/d4ra05531d)
Supplement: RA-014-D4RA05531D-s001 [file RA-014-D4RA05531D-s001.pdf]

Supplementary Information (SI) for RSC Advances.
This journal is © The Royal Society of Chemistry 2024

Efficient adsorption performance of uranium in wastewater by novel MXene material $TiVCT_x$ and its aerogel composites

Xiaoxia Luo[a,*], Xianliang Ren[b], Hongwei Wang[b,**]

[a] Chongqing College of Mobile Communication, Chongqing, 401520, P.R. China

[b] National Key Laboratory of Advanced Casting Technologies, Chongqing Key Laboratory of Soft Condensed Matter Physics and Smart Materials, College of Physics, Chongqing University, Chongqing, 400044, P.R. China

E-mail addresses:20202701013@cqu.edu.cn (H. Wang); xiaoxialuo90@163.com (X. Luo)

Table S1 Mocledn's artificial seawater formula (salinity 3.34%)

Chemical formula	Concentration (g L^{-1})
NaCl	26.726
$MgCl_2$	2.26
$MgNO_3$	5.845
$CaCl_2$	1.153

Adjusting the pH of a 100 mL, 125 mg/L U(VI) solution solely with NaOH reveals minimal changes in U(VI) concentration across varying pH levels. Only a negligible amount of U(VI) precipitates, hence its effect on adsorption can be disregarded.

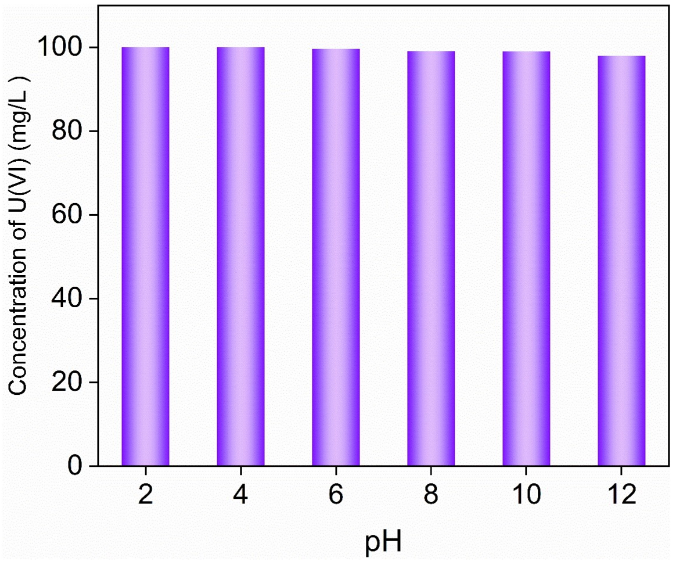

Fig. S1 The influence of NaOH-adjusted pH on U(VI) concentration.
